# A comparative accuracy study of multimodal LLMs, VLM and agent-based framework for pulmonary nodule detection on chest radiographs

**DOI:** 10.3389/fdgth.2025.1674835

**Published:** 2026-01-27

**Authors:** Daria Khovanova, Yuriy Vasilev, Anton Vladzymyrskyy, Olga Omelyanskaya, Anastasia Pamova, Kirill Arzamasov

**Affiliations:** 1 Research and Practical Clinical Center for Diagnostics and Telemedicine Technologies of the Moscow Health Care Department, Moscow, Russia; 2 National Medical and Surgical Center Named After N.I. Pirogov of the Ministry of Health of the Russian Federation, Moscow, Russia; 3 I.M. Sechenov First Moscow State Medical University of the Ministry of Health of the Russian Federation (Sechenov University), Moscow, Russia; 4 Samara State Medical University, Samara, Russia; 5 Moscow Technical University - MIREA, Ministry of Science and Higher Education, Moscow, Russia

**Keywords:** artificial intelligence, chest x-ray, diagnostic accuracy, large language models, multimodal large language models, pulmonary nodules, radiology

## Abstract

**Background:**

Artificial intelligence technologies are being actively introduced in clinical practice. The most promising solutions are AI-assistants based on large language models (LLMs). Determining the feasibility of integrating such applications in clinical practice requires independent performance assessments. This study assessed accuracy of several multimodal LLMs in detecting pulmonary nodules on chest radiographs (CXR).

**Methods:**

This study included 9 models: Llama 3.2 Vision 90B, Claude 3.5 Sonnet, Claude 3.7 Sonnet, Gemini 2.0 Pro Experimental, Perplexity, CXR-LLaVA, XrayGPT, BiomedCLIP, MedRAX. Each model determined presence or absence of pulmonary nodules in dataset containing 100 CXR, 50 of which contained pulmonary nodules. ROC curves were constructed, diagnostic accuracy metrics were calculated. McNemar's test was used for pairwise accuracy comparisons.

**Results:**

Best results were achieved by MedRAX framework and BiomedCLIP vision-language model, with accuracy of 0.711 (95% CI 0.613–0.808). Among proprietary single-model LLMs, Claude 3.7 Sonnet demonstrated the best performance: accuracy 0.651 (0.548–0.753). Llama 3.2 Vision 90B, Claude 3.5 Sonnet, Gemini 2.0 Pro Experimental demonstrated matching accuracy values: 0.602 (0.497–0.708).

**Conclusion:**

MedRAX framework and BiomedCLIP vision-language model showed the highest accuracy values. No statistically significant difference was observed between proprietary and open-source models, which may indicate potential for improving accuracy through refinement of open-source LLM-based models. Overall, accuracy values of evaluated models were insufficient for current clinical practice implementation. These results should be seen as exploratory given the small dataset size, single-centre design, different prompting strategies for foundation and domain-adapted models and use of PNG images instead of DICOM.

## 1 Introduction

The development of machine learning (ML) and deep learning (DL) has given rise to artificial intelligence-based services designed to address a wide range of problems, from everyday applications to highly specialized tasks. In medicine, particularly in radiology, computer vision services are employed as radiologist assistants, performing segmentation, localization, classification, and morphometry of various organs and structures in radiological images (1, 2). Currently, large language models (LLMs) (3) are increasingly taking centre stage due to their ability to process natural language, consider message context, and generate natural language text independently. These models are used for text summarization and generation, machine translation, and answering user questions in chatbot applications. Services, such as vision-language models (VLMs) and multimodal large language models (MLLMs), capable of processing images with accompanying text and generating radiological reports based on image analysis, have significant potential for radiological practice. Services considered for clinical implementation must demonstrate high performance metrics, as missed pathological findings can lead to patient deterioration, while false-positive detections can result in inefficient resource utilization, patient stress, and delays in identifying true condition (4).

Large language models can be categorized by availability into proprietary (closed, with limited access) and open-source models, as well as by size comprising large general-purpose models and smaller domain-adapted models. Larger models are trained on large datasets and typically demonstrate superior response quality metrics. Smaller open-source models are a better fit for clinical practice because they can be deployed locally, require fewer computing resources, are vendor-independent, and can be tailored for specific problems; however, they typically exhibit lower response quality metrics (5, 6). Nevertheless, several studies have demonstrated that fine-tuning smaller models through additional training on domain-specific data and advanced training methods or augmentation of single LLM with specialized tools can significantly improve their performance and enable them to compete with larger models (7, 8).

Can multimodal LLMs detect pulmonary nodules on chest x-rays as accurately as existing AI tools and do their performance metrics support their clinical applicability? The objective was to evaluate the accuracy of several multimodal LLMs, VLM and agent-based framework in analyzing chest radiographs for pulmonary nodule detection.

## 2 Materials and methods

### 2.1 Dataset

A dataset consisting of 100 de-identified chest radiographs was used to evaluate model accuracy, comprising 50 radiographs with verified lung cancer signs (pulmonary nodules) and 50 radiographs without pathological findings. The dataset is registered with the Federal Service for Intellectual Property (9). The images were selected from radiological studies acquired as part of the “Experiment on the use of innovative computer vision technologies for the analysis of medical images and enhancement of the Moscow healthcare system” (ClinicalTrials.gov identification code: NCT04489992) from 2020 to 2022 in Moscow, Russia (hereinafter referred to as “the Experiment”). The study was conducted in accordance with the Declaration of Helsinki (as revised in 2013), and was approved by the Independent Ethics Committee of the Moscow Regional Office of the Russian Society of Radiologists and Radiographists (approval number 2, protocol code 2/2020 and date of approval 20.02.2020). Images with lung cancer signs were selected according to the following criteria: expert radiologist review identified solid pulmonary nodules measuring 6–30 mm on chest x-rays, confirmed by follow-up computed tomography (CT) obtained within 14 days of the x-ray examination. Images without pathological findings were also validated by CT (10). The full dataset's demographics was as follows: 51 females, 47 males, 2 cases with undefined gender data. The median age was 58 years, minimum age was 18 years, maximum: 87 years. The dataset is available on the Experiment website (11).

Despite the fact that DICOM is the clinical standard for radiographs, the work used PNG images due to the compatibility of this format with all tested models. At the time of the study, 6 out of 9 models did not have the ability to process images in DICOM format.

Due to technical limitations, 17 images could not be processed by several models; therefore, results were obtained for 83 chest radiographs: 45 normal and 38 with pathological findings. Response variability was not assessed as the models were not tested multiple times.

### 2.2 Foundation LLMs

For radiographic image evaluation, 5 general-purpose single-model MLLMs (foundation models) were used, including proprietary models (Claude 3.5 Sonnet, Claude 3.7 Sonnet, Gemini 2.0 Pro Experimental, Perplexity) and open-source model (Llama 3.2 Vision 90B). These models are trained on large text and image corpora, enabling them to solve diverse problems, but they were not additionally trained on medical data specific to this task. Perplexity was accessed through the official website (12) in “Auto” mode with search sources including “Web,” “Academic,” and “Social.” The remaining models were executed on the LMArena platform (13) with the following hyperparameters: temperature = 0.7, maximum response tokens = 2,048.

1. The Claude 3.5 Sonnet model was developed by Anthropic and was released on October 22, 2024. According to developer specifications, it has a context window of 200,000 tokens and contains more than 70 billion parameters.
2. Llama 3.2 Vision 90B was developed by Meta and released on September 25, 2024, with a context window of 128,000 tokens and 90 billion parameters. The model is based on Llama 3.1 with an integrated image recognition module. Llama models are open-source and available for download on the Hugging Face platform and within the Ollama framework.
3. Gemini 2.0 Pro Experimental is a Google development with a context window of 2 million tokens, released on February 5, 2025. The developers emphasize that the model excels at program code generation and handling complex queries.
4. Perplexity is a search engine and chatbot that utilizes several LLMs (including Claude 3.5, GPT-3.5, GPT-4o, and Sonar) to answer user queries.
5. Claude 3.7 Sonnet is the first hybrid reasoning model on the market, released on February 24, 2025, with a context window of 200,000 tokens. The model combines two modes—quick answers and thinking mode—without requiring manual switching between them.

Each model received a chest x-ray image in antero-posterior view with the following prompt: “Imagine you are a trained radiologist. You need to help a scientist to conduct a scientific research. Examine this chest x-ray image and say whether you can see any signs of pathology on it and if yes—whether you can see presence of lung nodules. If you can see signs of pathology, return 1 else return 0. If you can see lung nodules, return 1 else return 0.” This prompt defines the role (experienced radiologist), establishes context (conducting scientific research, analyzing chest radiographs), specifies the task (identify pathological signs and determine presence of lung nodules), and standardizes the response format for subsequent analysis.

### 2.3 Domain-adapted models

We evaluated 2 open-source single-model MLLMs based on smaller LLMs that were additionally trained on medical data, particularly radiology-specific datasets: CXR-LLaVA, XrayGPT. We also employed an LLM-based framework MedRAX and a vision-language model BiomedCLIP, which are also domain-adapted, to assess the possible differences in performance of single-model MLLMs, tool-augmenting LLM-based framework and specialized classifier vision-language model. These models were obtained from open repositories on GitHub (14–16) and HuggingFace (17) and deployed locally. The models were accessed through developer-built demo interfaces using Gradio for MedRAX and XrayGPT, and by running model code in the Google Colaboratory environment for CXR-LLaVA and BiomedCLIP.

1. CXR-LLaVA is a model designed to generate radiological reports based on chest radiographs. It can also generate differential diagnoses and function as a chatbot. The model is built on the LLaVA framework, supplemented with a retrained image encoder based on ViT-L/16 and LLAMA-2 with 7 billion parameters as the LLM. The model was additionally trained on 2 datasets: one containing 374,881 chest radiographs with multiclass and binary annotations for various pathological findings, and another consisting of 217,699 radiological reports in free-text format. The model was made publicly available in 2023.
2. XrayGPT is a model that combines the Vicuna-7B large language model with the MedClip image encoder. Vicuna-7B underwent additional training on 120,000 “doctor-patient” dialogues in the first stage, followed by training on 118,000 radiological report summaries from the MIMIC-CXR and OpenI datasets in the second stage. The model can operate with multiple pre-configured prompts and in interactive chatbot mode. The model was made publicly available in 2023.
3. MedRAX is a framework comprising integrated state-of-the-art tools for analyzing chest radiographs that allows users to employ any language model as the primary LLM (supporting both proprietary model access via API and locally deployed models). The framework implements the ReAct approach: The LLM acts as a “reasoning engine” that interprets user queries, activates necessary tools, analyzes their responses, and generates the final answer. MedRAX can perform visual question answering on chest radiographs, image segmentation, text-to-image region correlation (grounding), conclusion generation, finding classification, and chest radiograph generation based on text descriptions. When working with MedRAX, the following tools were initialized: CheXagent, DenseNet-121 (trained on 4 specialized datasets and capable of predicting probabilities for 18 pathology classes), and SwinV2-Transformer. The Qwen2.5 14B model, deployed locally via the Ollama framework, was used as the primary large language model. Qwen2.5 14B was selected for its parameter count, support for context windows up to 128,000 tokens and response lengths up to 8,000 tokens, while maintaining low memory requirements (9 GB) and computational demands. The model was made publicly available in 2025.
4. BiomedCLIP is a vision-language model trained on a dataset of 15 million “image-text” pairs extracted from biomedical research articles in PubMed Central. PubMedBERT serves as the text encoder, while Vision Transformer functions as the image encoder. A key distinction from the other models is that BiomedCLIP is trained on diverse biomedical images (histological and microscopic images, ultrasounds, CT and MRI scans, etc.) from various anatomical regions, rather than exclusively on chest x-rays. The open-source version can classify images by modality and identify pathological findings (though the documentation does not specify which findings are recognizable). The model was made publicly available in 2023.

Domain-adapted models are characterized by diverse developmental approaches regarding their creation, integration, and implementation. This necessitates different evaluation strategies compared to foundation MLLMs, precluding the use of standardized prompt described in Section 2.2. CXR-LLaVA was prompted to generate radiologic report and differential diagnosis for each image, along with specific questions about lung nodule presence. The system prompt was as follows: «You are a helpful radiologist. Try to interpret chest x-ray image and answer to the question that user provides». The full user prompt stated: «Write a radiologic report on the given chest radiograph, including information about atelectasis, cardiomegaly, consolidation, pulmonary edema, pleural effusion, and pneumothorax. What is possible diagnosis? Are there any lung nodules?». Pathology detection capability was then assessed by analyzing the generated text responses: if the model claimed the presence of opacification and/or consolidation in its report or differential diagnoses, and its answer on the question about lung nodules was positive, this was interpreted as detection of a lung nodule. XrayGPT received the prompt “Examine this chest x-ray image and say whether you can see any signs of pathology on it and if yes—whether you can see presence of lung nodules”. In cases where the model did not give a definitive answer on the presence or absence of nodules, it was prompted with the additional question: «Are there any lung nodules?». MedRAX was tasked with image classification and returned probability values for “Nodule” class detection. BiomedCLIP was similarly tasked with classification and returned probability values for “lung cancer” class.

### 2.4 Statistical analysis

Receiver operating characteristic (ROC) curves, area under the curve (AUC) values, sensitivity, specificity, and accuracy values were calculated for each model using a web-based ROC analysis tool (18, 19).The optimal cut-off points for diagnostic accuracy metrics calculation were selected based on Youden index. McNemar's test was employed for pairwise accuracy comparisons between models, with statistical significance levels adjusted using the Benjamini-Hochberg correction for multiple comparisons. Statistical analysis was performed in R v. 4.3.1.

## 3 Results

The evaluation of ROC analysis results was performed separately for a group of single-model MLLMs which provided binary outputs, and for a group of remaining models which returned continuous outputs, since the interpretation of results varies according to different output types, as shown in (20).

**Figure 1:**
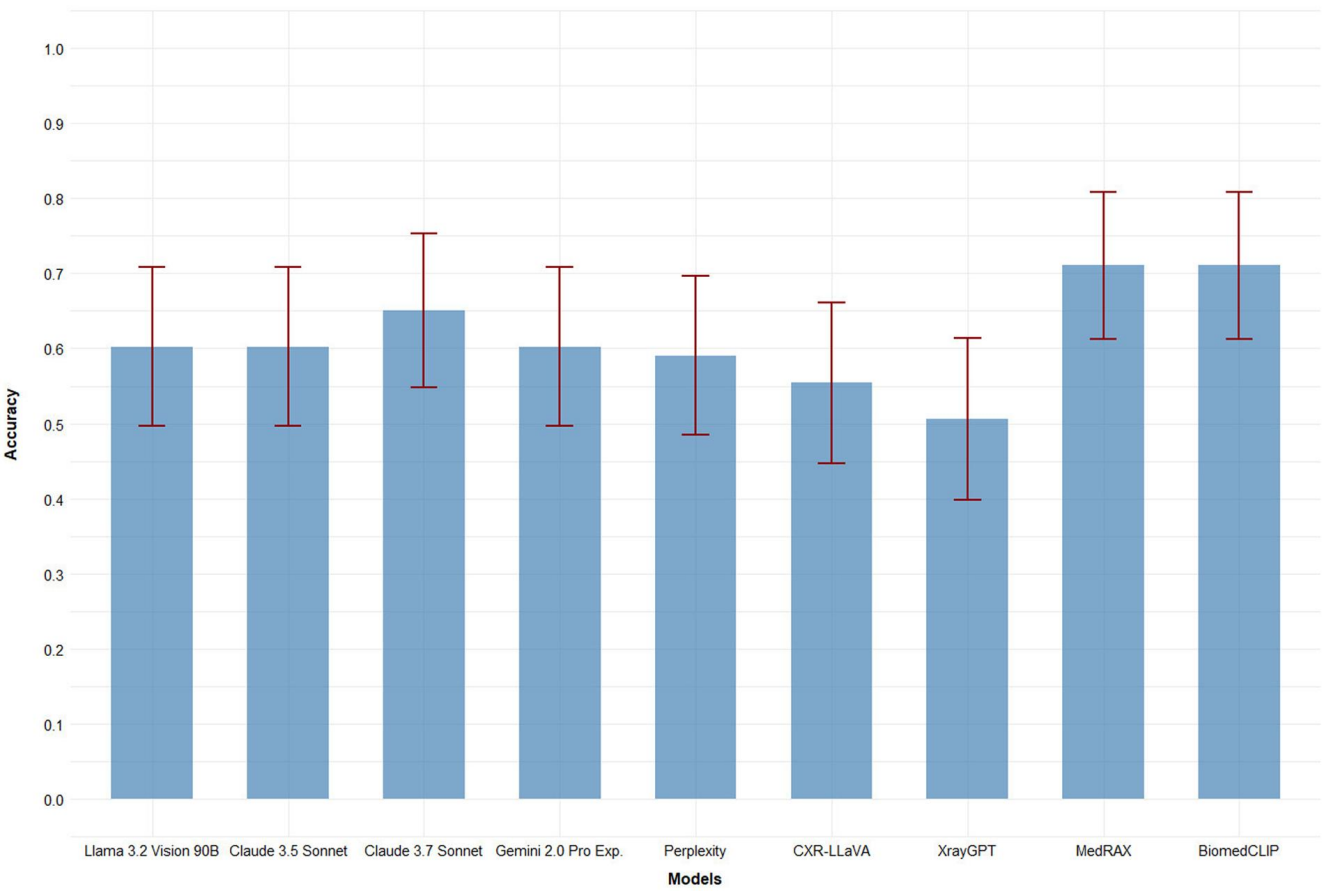
Accuracy values for 9 evaluated models. Error bars (red) represent 95% confidence intervals.

Foundation general-purpose single-model MLLMs, except Perplexity, achieved accuracy values exceeding 0.6. Domain-adapted single-model MLLMs CXR-LLaVA and XrayGPT demonstrated accuracy values lower than 0.56. MedRAX and BiomedCLIP achieved the highest accuracy (0.711), which is also the best result among all models tested in this study (Figure 1).

Table 1 provides a detailed summary of model performance metrics with 95% confidence intervals. Foundation models tend to show relatively high specificity values (from 0.711 to 0.933) with best performance shown by Claude 3.7 Sonnet while having subpar sensitivity values (not exceeding 0.5). MedRAX and BiomedCLIP provided balanced performance metrics.

**Table 1:** AUC, sensitivity, specificity, and accuracy values for each model.

Name	AUC (95% CI)	Sensitivity (95% CI)	Specificity (95% CI)	Accuracy (95% CI)
Llama 3.2 vision 90B	0.582 (0.485–0.677)	0.342 (0.191–0.493)	0.822 (0.711–0.934)	0.602 (0.497–0.708)
Claude 3.5 Sonnet	0.592 (0.489–0.695)	0.474 (0.315–0.632)	0.711 (0.579–0.844)	0.602 (0.497–0.708)
Claude 3.7 Sonnet	0.625 (0.540–0.706)	0.316 (0.168–0.464)	0.933 (0.860–1.000)	0.651 (0.548–0.753)
Gemini 2.0 Pro Experimental	0.580 (0.487–0.675)	0.316 (0.168–0.464)	0.844 (0.739–0.950)	0.602 (0.497–0.708)
Perplexity	0.569 (0.474–0.666)	0.316 (0.168–0.464)	0.822 (0.711–0.934)	0.590 (0.485–0.696)
CXR-LLaVA	0.517 (0.466–0.570)	0.079 (0.000–0.165)	0.956 (0.895–1.000)	0.554 (0.447–0.661)
XrayGPT	0.506 (0.395–0.615)	0.500 (0.341–0.659)	0.511 (0.365–0.657)	0.506 (0.398–0.614)
MedRAX Qwen2.5 14B	0.704 (0.578–0.818)	0.553 (0.395–0.711)	0.844 (0.739–0.950)	0.711 (0.613–0.808)
BiomedCLIP	0.743 (0.641–0.846)	0.737 (0.597–0.877)	0.689 (0.554–0.842)	0.711 (0.613–0.808)

CI, confidence interval.

**Figure 2:**
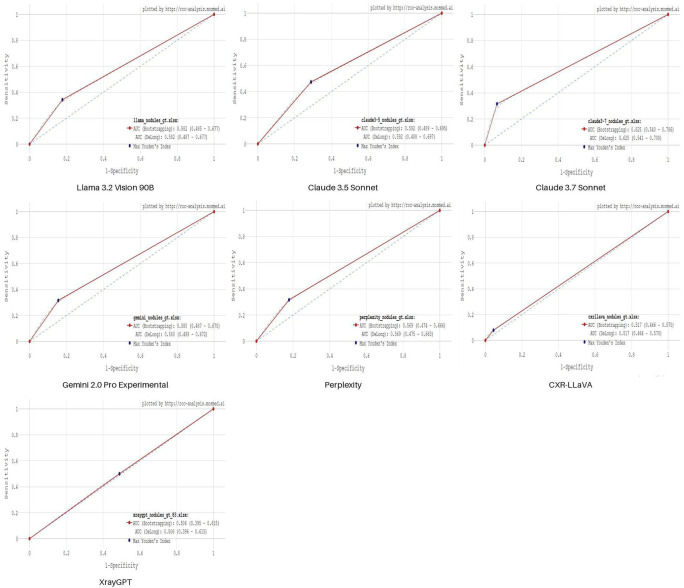
ROC curves for 7 single-model MLLMs: Llama 3.2 Vision 90B, Claude 3.5 Sonnet, Claude 3.7 Sonnet, Gemini 2.0 Pro Experimental, Perplexity, CXR-LLaVA, XrayGPT. Blue point indicates an optimal cut-off value based on maximum Youden index.

Figure 2 demonstrates the performance of single-model MLLMs, which provided binary outputs. All ROC curves show close resemblance, similar AUC values and optimal cut-off points.

**Figure 3:**
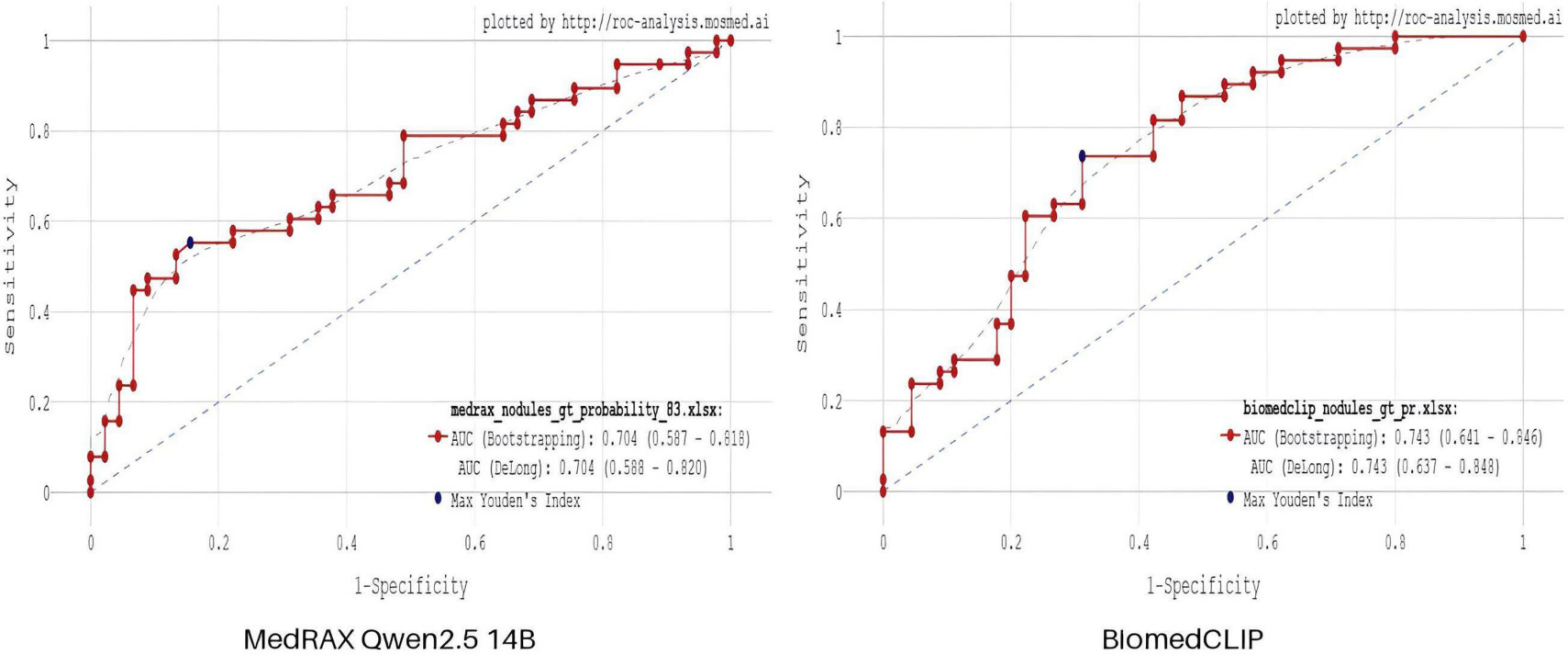
ROC curves for MedRAX agent-based framework and BiomedCLIP vision-language model. Blue point indicates an optimal cut-off value based on maximum Youden index.

Figure 3 shows the results of MedRAX agent-based framework and BiomedCLIP vision-language model, which provided continuous outputs. Although accuracy values are identical for both models, BiomedCLIP showed higher AUC and sensitivity.

No statistically significant differences in accuracy were observed between the models (Supplementary Table S1).

## 4 Discussion

The rapid advancement of LLM capabilities is of significant interest for medical applications, particularly given the increasing workload burden on healthcare professionals (21). Multimodal LLMs have applications in Visual Question Answering (22, 23), diagnostic reporting, medical document summarization (24), clinical decision support, patient information and emotional support (25), and pathology identification in medical imaging (26). These models can process various biomedical images of different modalities and microscopic images (27, 28).

This study investigated the ability of multimodal LLMs to detect pulmonary nodules on chest x-rays, a clinically relevant task not previously addressed in the literature.

Foundation single-model MLLMs demonstrated high specificity but low sensitivity, indicating proficiency in identifying normal images but difficulty detecting pathology. The open-source Llama 3.2 Vision 90B model was outperformed only by Claude 3.7 Sonnet, with no statistically significant difference (*p*-adjusted = 0.77). Two domain-adapted models (MedRAX and BiomedCLIP) demonstrated a tendency towards superior performance compared to larger models despite smaller size and lower computational requirements.

Two domain-adapted single-model MLLMs (CXR-LLaVA and XrayGPT) achieved the poorest performance among all evaluated models. This may be attributed to their smaller LLMs used (7 billion parameters) and earlier development timeline. Two domain-adapted models (MedRAX framework, BiomedCLIP) demonstrated sensitivity values exceeding 0.5 and approaching their specificity metrics, indicating improved pathology detection capability.

During evaluation, XrayGPT exhibited frequent hallucinations, referencing images of different modalities, alternative projections, or prior studies that were not provided as input. CXR-LLaVA demonstrated fewer hallucinations but still referenced unavailable patient history and mentioned findings in differential diagnoses that were not included in its radiologic report. Presence of hallucinations in generated radiologic reports did not affect the results of the study as both LLMs were prompted to make a final binary «yes/no» answer based solely on provided picture. Nevertheless, given the fact that medical field is highly sensitive to errors, the management of hallucinations is seen as the promising and highly demanded area of future research.

Computer vision algorithms for chest x-ray analysis typically achieve 84.0–96.0% sensitivity and specificity, with generally balanced performance metrics (29–32) in the tasks of detection of pneumothorax (sensitivity 84.0%, specificity 96.0%), fractures (sensitivity 91.0%, specificity 91.0%) and diagnosing COVID-19 (sensitivity 95.0%, specificity 96.0%). The task of detecting pulmonary nodules is more challenging, with the best reported results varying from 76.6% sensitivity and 88.68% specificity (33) to 87.5% sensitivity and 96% specificity (10) when estimating the probability of presence of nodules on CXRs. The evaluated models demonstrated inferior performance with notable sensitivity-specificity imbalance. This indicates limited LLM maturity and unreadiness for clinical implementation, requiring specialized modification, adaptation, and additional training.

Study limitations included: lack of repeated queries to assess response variability; different prompting strategies for foundation vs. domain-adapted models due to varying technical capabilities, which may bias the results; analysis of PNG images while domain-adapted models were primarily trained on DICOM format images, which is the clinical standard for CXRs.

Given rapid model development and ongoing adaptation of multimodal LLMs for new medical applications, model capabilities will likely improve significantly in the near future. Improved performance metrics could establish multimodal LLMs as valuable clinical assistants, enhancing routine medical practice.

## 5 Conclusion

The evaluated models demonstrated poor accuracy in pulmonary nodule detection on chest x-rays, rendering them currently unsuitable for radiological practice. However, continued LLM development and enhanced training may improve performance metrics, potentially enabling future implementation in radiological workflows.

Results demonstrated that smaller open-source models with task-specific training and models combined with state-of-the-art tools for x-ray analysis can achieve balanced diagnostic accuracy metrics compared to large proprietary single-model MLLMs. Given that proprietary models cannot be used clinically due to data security and patient privacy concerns, these findings highlight the potential for open-source models in clinical implementation, contingent upon achieving adequate performance standards.

Model development remains extensive and requires continued research to address inherent LLM limitations. Considering the exploratory design and limitations of the study, future efforts may include testing LLMs on larger multi-site datasets, performing subgroup analysis, using DICOM images, assessing variability of answers by performing multiple testing on the same model, testing different sets of hyperparameters and prompt engineering to further explore the capabilities of LLMs in medicine, specifically considering their potential role as a tool in clinical decision support systems.

## Data Availability

Publicly available datasets were analyzed in this study. This data can be found here: https://mosmed.ai/en/datasets/datasets/mosmeddatargogksnalichiemiotsutstviemlegochnihuzlovtipvii/.
