# Supplementary material for: A comparative accuracy study of multimodal LLMs, VLM and agent-based framework for pulmonary nodule detection on chest radiographs
Source: Front Digit Health. 2026 Jan 27;7:1674835. doi: 10.3389/fdgth.2025.1674835 (PMC12887855; doi:10.3389/fdgth.2025.1674835)
Supplement: Supplementary file 1 [file Datasheet1.pdf]

Supplementary Material

Supplementary Tables

Supplementary Table S1: Pairwise comparison results: p-values adjusted using the Benjamini-Hochberg correction for multiple comparisons

	Llama 3.2 vision 90B	Claude 3.5 Sonnet	Claude 3.7 Sonnet	Gemini 2.0 Pro Experimental	Perplexity	CXR-LLaVA	MedRAX Qwen2.5 14B	Xray GPT	BiomedCLIP
Llama 3.2 vision 90B	-	0.93	0.77	0.93	1.00	0.77	0.48	0.57	0.48
Claude 3.5 Sonnet		-	0.77	0.93	1.00	0.81	0.48	0.57	0.77
Claude 3.7 Sonnet			-	0.77	0.77	0.47	0.77	0.47	0.77
Gemini 2.0 Pro Experimental				-	1.00	0.77	0.48	0.57	0.48
Perplexity					-	0.84	0.47	0.72	0.47
CXR-LLaVA						-	0.25	0.84	0.47
MedRAX Qwen2.5 14B							-	0.15	0.93
XrayGPT								-	0.15
BiomedCLIP									-
